# YWHAH activates the HMGA1/PI3K/AKT/mTOR signaling pathway by positively regulating Fra-1 to affect the proliferation of gastric cancer cells

**DOI:** 10.32604/or.2023.029698

**Published:** 2023-06-27

**Authors:** JUNYU HE, FENG ZENG, XI JIN, LIN LIANG, MENGXIANG GAO, WENTAO LI, GUIYUAN LI, YANHONG ZHOU

**Affiliations:** 1 NHC Key Laboratory of Carcinogenesis, Hunan Cancer Hospital and the Affiliated Cancer Hospital of Xiangya School of Medicine, Central South University, Changsha, 410013, China; 2 Cancer Research Institute, Basic School of Medicine, Central South University, Changsha, 410078, China; 3 Hunan Key Laboratory of Cancer Metabolism, Hunan Cancer Hospital and the Affiliated Cancer Hospital of Xiangya School of Medicine, Central South University, Changsha, 410013, China

**Keywords:** Gastric cancer, Fra-1, YWHAH, Signal transduction pathway, Cell proliferation

## Abstract

Fos-related antigen 1 (Fra-1) is a nuclear transcription factor that regulates cell growth, differentiation, and apoptosis. It is involved in the proliferation, invasion, apoptosis and epithelial mesenchymal transformation of malignant tumor cells. Fra-1 is highly expressed in gastric cancer (GC), affects the cycle distribution and apoptosis of GC cells, and participates in GC occurrence and development. However, the detailed mechanism of Fra-1 in GC is unclear, such as the identification of Fra-1-interacting proteins and their role in GC pathogenesis. In this study, we identified tyrosine 3-monooxygenase/tryptophan 5-monooxygenase activation protein eta (YWHAH) as a Fra-1-interacting protein in GC cells using co-immunoprecipitation combined with liquid chromatography-tandem mass spectrometry. Experiments showed that YWHAH positively regulated Fra-1 mRNA and protein expression, and affected GC cell proliferation. Whole proteome analysis showed that Fra-1 affected the activity of the high mobility group AT-hook 1 (HMGA1)/phosphatidylinositol-4,5-bisphosphate 3-kinase (PI3K)/protein kinase B (AKT)/mechanistic target of rapamycin (mTOR) signaling pathway in GC cells. Western blotting and flow cytometry confirmed that YWHAH activated HMGA1/PI3K/AKT/mTOR signaling pathway by positively regulating Fra-1 to affect GC cell proliferation. These results will help to discover new molecular targets for the early diagnosis, treatment, and prognosis prediction of GC.

## Introduction

Globally, gastric cancer (GC) is the fifth most common malignant tumor and the third leading cause of cancer-related mortality [1–5]. Its pathogenesis is a multi-factor and multi-step process, involving infection by microorganisms such as *Helicobacter pylori*, the activation of proto oncogenes such as *FOSL1* (encoding Fos-related antigen 1, also known as Fra-1), *BRD4* (encoding bromodomain containing 4), and *THY1* (encoding Thy-1 cell surface antigen, also known as CD90), and the inactivation of tumor suppressor genes [6–12]. *Helicobacter pylori* is considered to be a major cause of GC, but not in all cases [12–16], and the mechanism of GC is not completely clear. Therefore, more experimental evidence is needed to clarify its pathogenesis and potential molecular targets.

Fra-1 is a member of the activator protein-1 (AP-1) transcription factor superfamily and Fos family of proteins, and its basic domain is highly homologous with c-Fos [17,18]. Fos family proteins encode leucine zipper proteins that can form dimers with JUN family proteins to form the transcription factor complex AP-1 [18]. Fra-1 is an important nuclear transcription factor that regulates the growth, differentiation, and apoptosis of normal cells. It is abnormally expressed in many cancer cells and tissues, and plays an important role in the tumorigenesis and progression or maintenance of many tumor types [6,7,19–21]. Fra-1 levels are frequently elevated in a variety of human cancers because of carcinogenic signal transduction, and Fra-1 is closely related to metastasis and poor prognosis [20–22]. Overexpression of Fra-1 can lead to morphological changes of fibroblasts and is related to mesenchymal characteristics and E-cadherin downregulation in cancer cells [23–25]. In the cell cycle, Fra-1 is recruited to the promoter of *CCNA2* (encoding cyclin A2). Fra-1 induces the expression of Jun B, which interacts with the *CCNA2* promoter to enhance cell proliferation [26,27]. Previous studies suggest that Fra-1 plays an important role in the occurrence and development of malignant tumors such as GC.

In a previous study, we used quantitative real-time polymerase chain reaction (qPCR), immunohistochemistry (IHC), and western blotting to show that Fra-1 is highly expressed in GC tissues. *In vitro* experiments confirmed that Fra-1 overexpression inhibited the apoptosis of GC cells, increased the proportion of S-phase cells, and was related to an imbalance of the phosphatidylinositol-4,5-bisphosphate 3-kinase (PI3K)/protein kinase B (AKT) signaling pathway [6]. However, the detailed mechanism of Fra-1 in GC has not been fully defined. In this study, we aimed to identify molecules that interact with Fra-1 in GC cells using co-immunoprecipitation (Co-IP) combined with liquid chromatography-tandem mass spectrometry (LC-MS/MS), and clarified their mutual regulation relationship. Then, western blotting and flow cytometry were used to confirm that the newly identified Fra-1 interacting molecule, tyrosine 3-monooxygenase/tryptophan 5-monooxygenase activation protein eta (YWHAH), could promote the proliferation of GC cells by positively regulating Fra-1 to activate the high mobility group AT-hook 1 (HMGA1)/PI3K/AKT/mechanistic target of rapamycin (mTOR) signaling pathway. Our results provide a new molecular target to explore the pathogenesis of GC.

## Materials and Methods

### Cell lines and plasmids

Gastric cancer cells SGC7901 and AGS were preserved and provided by the Cancer Research Institute, Basic School of Medicine, Central South University. The cells were cultured via adherent growth in an incubator set at 37°C with 5% CO_2_ in Roswell Park Memorial Institute (RPMI) 1640 medium (Gibco-Life Technologies at Thermo Fisher Scientific, Waltham, MA, USA) with 10% fetal bovine serum (FBS; Gibco-Life Technologies) and 1% Penicillin-Streptomycin Solution.

The plasmids used in this study included: vector pLVX-mCMV-ZsGreen-PGK-Puro-Fra-1 (Fra-1 overexpression), vector pLVX-mCMV-ZsGreen-IRES-Puro-YWHAH (YWHAH overexpression), vector pLVX-mCMV-ZsGreen-PGK-Puro-Fra-1-D1 (expressing Fra-1 aa 1–107), vector pLVX-mCMV-ZsGreen-PGK-Puro-Fra-1-D2 (expressing Fra-1 aa 1–127), and vector pLVX-mCMV-ZsGreen-PGK-Puro-Fra-1-D3 vector (expressing Fra-1 aa 107–271). All plasmids were lentivirus expression vectors carrying an ampicillin resistance gene. Puromycin was used for screening.

### Immunoprecipitation

After transfection, the cells were cultured in a 100 mm dish for 48 h and then lysed using IP lysis buffer containing a mixture of protease inhibitors and phosphatase inhibitors (Beyotime Biotechnology, P0013, Shanghai, China). Then, 1 µg of primary antibody was incubated with 30 µL of protein A+G beads on a rotator at room temperature for 2 h, the protein lysate was then added and incubated on the rotator at 4°C overnight. Next day, the beads and immune complexes were washed five times with IP lysis buffer by rotating at room temperature for five minutes at a speed (2000 rpm/min) of 20 s per round. The sample was added to the corresponding SDS loading buffer and heated at 95°C for 5 min. The immunoprecipitated samples were detected using western blotting.

### Mass spectrometry analysis

The purified protein complex eluent was concentrated using IP beads, separated by SDS-PAGE, and stained with Coomassie brilliant blue (Beyotime Biotechnology). The corresponding protein band was excised from the gel, reduced, alkylated, and digested overnight using trypsin at 37°C (Thermo Fisher Scientific, Waltham, MA, USA). The digested peptides were dried and resuspended in MS compatible buffers, and the mixture was analyzed using an LTQ Orbitrap velos MS instrument (Thermo Fisher Scientific) in combination with a UltiMate RSLC Nano LC system (Dionex, Sunnyvale, CA, USA). Proteome Discoverer 1.4 software (Thermo Fisher Scientific) was used to identify proteins, import files, and search the UniProtKB/Swiss-Prot databases. The mass tolerances of precursors and fragments were set to 10 ppm and 0.8 Da, respectively. Peptide data with an error detection rate of <1% (*p* < 0.01) were discarded.

### RNA extraction and quantitative real-time reverse transcription PCR (qRT-PCR)

The total RNA of GC cells was isolated using the TRIzol reagent (Invitrogen, Waltham, MA, USA), and cDNA synthesis was carried out using the RevertAid First Strand cDNA synthesis kit (CWBio) according to the manufacturer’s facturer’s recommendations. qRT‑PCR was carried out with GoTaq qPCR Master Mix (Promega, Fitchburg, WI, USA). For detection of *Fra-1, YWHAH, AKT, PI3K, PDK1, MTOR*, and *HMGA1* mRNA expression levels, GAPDH was amplified in parallel as an internal control. The sequences of the primers used for qPCR were as follows: *Fra-1* forward 5′-CAGTGGATGGTACAGCCTCATTTC-3′, reverse 5′-GCAGTCTCCTGTTCACAAGGC-3′; *YWHAH* forward 5′-TCAAGAAGGTGGTGAAGCAGG-3′, reverse 5′-TCAAAGGTGGAGGAGTGGGT-3′; *AKT* forward 5′-ACACCAGGTATTTTG ATGAGGAG-3′, reverse 5′-TCAGGCCGTGCCGCTGGCCGAGTAG-3′; *PI3K* forward 5′-AGCTGGTTTGGATCTTCGGA-3′, reverse 5′-CAGGTC ATCCCC AGAGTTGT-3′; *PDK1* (encoding pyruvate dehydrogenase kinase 1) forward 5′-AGTTCATGTCACGCTGGGTA-3′, reverse 5′-CAGCTTCAGGTCTCCTTGGA-3′; *MTOR* forward 5′-GCAACCCTTCTTTGACAACATTTTT-3′, reverse 5′-ATTTCTTCTCTCAGACGCTCTCC-3′; *HMGA1* forward 5′-TGCGAAGAAACTGGG AGAGA-3′, reverse 5′-TGCGAAGAAACTGGGAGAGA-3′; *GAPDH* (encoding glyceraldehyde-3-phosphate dehydr-ogenase) forward 5′-TCAAGAAGGTGGTGAAGCAGG-3′, reverse 5′-TCAAAGGTGGAGGAGT GGGT-3′. The expression of mRNA was assessed by evaluated threshold cycle (CT) values. The CT values were normalized to the expression levels of GAPDH and the relative amount of mRNA specific to each of the target genes was calculated using the 2^−ΔΔCT^ method. qPCR was carried out using the Bio‑Rad CFK96™ Real‑Time System (Bio‑Rad, Hercules, CA, USA). The data were analyzed by Bio‑Rad CFK Manager software (Bio‑Rad).

### siRNA experiments

To test the effect of silencing *YWHAH* and *Fra-1*, we selected three small interfering RNAs (siRNAs) targeting different regions of the *YWHAH* and *Fra-1* genes. The three siRNA sequences for *YWHAH* were:

si-YWHAH-001 5′-GCUGGAGACAGUUUGCAAUTT-3′;

si-YWHAH-002 5′-CCAGAAUGCACCUGAGCAATT-3′;

si-YWHAH-003 5′-GCUGAGCUGGACACACUAATT-3′;

and the control si-NC 5′-ACGUGACACGUUCGG AGAATT-3′.

The three siRNA sequences for *Fra-1* were:

si-Fra-1-001 5′-GTCGAAGGCCTTGTGAA-3′;

si-Fra-1-002 5′-GCTCATCGCAAGAGTAGCA-3′;

si-Fra-1-003 5′-GGAAGGAACTGACCGACTT-3′;

and the control si-NC 5′-ACGUGACACGUUC GGAGA ATT-3′.

For siRNA transfection, the riboFECT CP Transfection Kit (Guangzhou RiboBio Co., Ltd., Guangzhou, China) was used according to the manufacturer’s instructions, and then the silencing effect was detected. The most successful siRNA sequence for each gene was used for subsequent experiments.

### 5-ethynyl-2′-deoxyuridine (EdU) cell proliferation assay

Cells (1 × 10^5^ to 3 × 10^6^) were inoculated in 6-well plates and cultured at 37°C in a 5% CO_2_ incubator to the required cell density. Two hours before transfection, serum-free 1640 medium was used. The transfected cells were divided into groups according to the corresponding experiments. After 6 h, the mixed solution was sucked out and replaced with normal culture medium, and culture was continued for 48 h. Referring to the operating instructions of the EdU-647 cell proliferation test kit (Beyotime biotechnology, C0081S), the samples were processed before flow cytometry [28,29]. Finally, the samples were tested by flow cytometry at a 488 nm excitation wavelength and 520 nm emission wavelength. Cells were analyzed with Moflo XDP High-Performance Cell Sorter (Beckman Coulter). Data were acquired and analyzed with Summit v5.2 software.

### Protein extraction and western blotting

Cells were digested with trypsin and then lysed with Radioimmunoprecipitation assay (RIPA) buffer (CWBio, Beijing, China). The sample was centrifuged at 12000 × *g* at 4°C for 15 min to remove insoluble matter. The protein concentration of the retained supernatant was detected, and protein electrophoresis was carried out using a 10% SDS-PAGE gel at 50 V for 40 min, and then at 120 V for 60 min (PowerPac Universal, Bio-Rad, Hercules, CA, USA). Then, the proteins were transferred to a polyvinylidene fluoride (PVDF) membrane (HyClone Laboratories, Logan, UT, USA) at 100 V for 90 min, and then incubated in phosphate-buffered saline (PBS)-Tween20 with 5% skimmed milk for 1–2 h. The membrane was then incubated with primary antibody at 4°C overnight.

The main antibodies used in this study include: Fra-1 (YM0122,Immunoway, Plano, TX, USA), YWHAH (ab206292, Abcam, Cambridge, MA, USA), HMGA1 (#7777, Cell Signaling Technology, Danvers, MA, USA), PI3K (AF6241, Affinity Biosciences, Cincinnati, OH, USA), AKT (10176-2-AP, Wuhan Sanying Biotechnology Co., Ltd., Wuhan, China), mTOR (ab32028, Abcam), phosphorylated (p)-mTOR (ab109268, Abcam), GAPDH (AB-P-R001, Hangzhou Xianzhi Biotechnology Co., Ltd., Hangzhou, China). The dilution ratio of the antibodies was 1:1000. Rabbit anti-β-tubulin antibody, mouse anti-β-actin, Rabbit anti-GAPDH, from Affinity Bioscience were used at a dilution of 1:5000; anti-rabbit secondary antibody and anti-mouse secondary antibody were purchased from Lianke company (Shanghai, China), and used at a dilution of 1:3000. The membranes were incubated with the secondary antibodies for 1 h and then the immunoreactive proteins were visualized using Molecular Imager Gel Dox XR System (Bio-Rad Laboratories).

### Statistical analysis

GraphPad Prism 5 software (GraphPad Inc., La Jolla, CA, USA) was used perform the statistical analyses. T-tests were used to analyze whether the difference between any two groups was significant. One way analysis of variance (ANOVA) was used to analyze whether the data of more than two groups were significantly different. All experiments were set with three or more replicates, and *p* < 0.05 indicated a significant difference.

## Results

### Identification of YWHAH as an interacting protein of Fra-1 in GC cells by Co-IP and LC-MS/MS technologies

**Figure 1:**
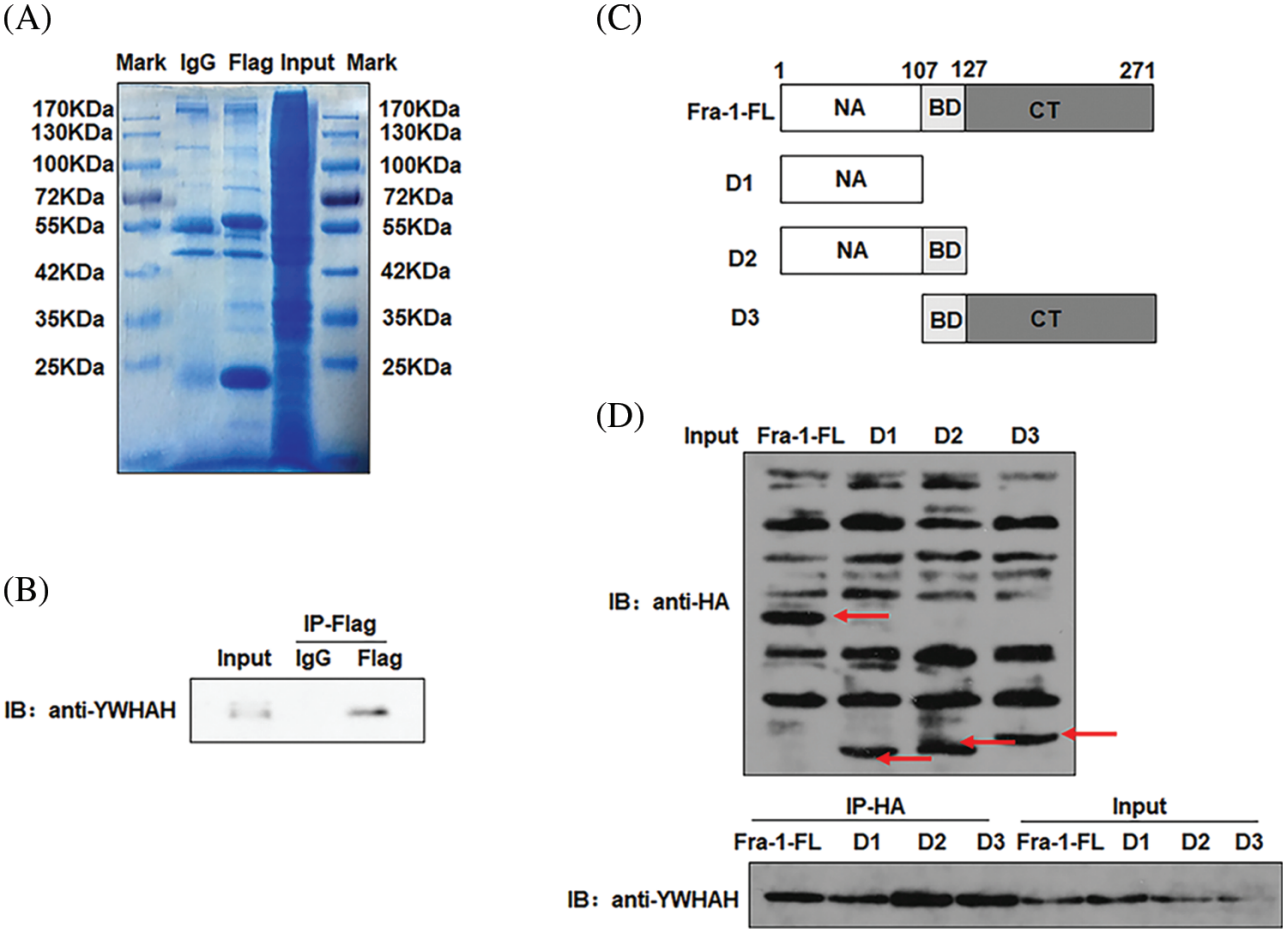
Identification of new interacting molecules of Fra-1 in SGC7901 GC cells. (A) Staining diagram of FLAG-Fra-1 precipitated using anti-FLAG affinity gel beads and its interacting proteins. Input lane: the whole cell protein as the positive control; IgG Lane: the protein bound to IgG as the negative control; FLAG lane: proteins binding to Fra-1. (B) Western blotting showing that there was an endogenous interaction between YWHAH and Fra-1 in SGC7901 GC cells. Input lane: the whole cell protein as the positive control; IgG Lane: the protein bound to IgG as the negative control; FLAG lane: proteins binding to Fra-1. (C) Schematic diagram of Fra-1 domains. Fra-1-FL: the full length amino acid sequence of Fra-1, D1 (1–107 aa), D2 (1–127 aa), and D3 (107–271 aa) domains of Fra-1. (D) The interaction domain between YWHAH and Fra-1 was detected by western blotting. Input Lane: the whole cell protein as the positive control; IP-HA: an anti-HA antibody was used to detect the domain interacting with YWHAH. The experiment was performed three times.

Our previous research results showed that Fra-1 was upregulated in GC tissue and is involved in its occurrence and development. To systematically explore the role and possible mechanism of Fra-1 in GC, we first used Co-IP combined with LC-MS/MS to identify proteins that interact with Fra-1 in GC cells. After the Fra-1 interaction proteins were separated by SDS-PAGE, stained with Coomassie blue, and decolorized, we observed obvious difference bands at 25~40, 40~70, and 70~180 kDa on the gel (Fig. 1A). The bands were then analyzed by endogel enzymolysis and mass spectrometry. Among them, 25 specific proteins of the FLAG lane were identified at 25~40 kDa, among which 6 protein molecules belonged to the 14-3-3 protein family, including YWHAE, YWHAH, YWHAG, YWHAZ, and YWHAQ, suggesting that the 14-3-3 protein family might interact with Fra-1 (Suppl. Table 1). To verify the interaction between the 14-3-3 protein family and Fra-1, we performed IP experiments for YWHAE, YWHAH, YWHAG, YWHAZ, and YWHAQ with Fra-1, respectively. The results showed that Fra-1 interacted only with YWHAH in SGC7901 GC cells (Fig. 1B). To further explore the interaction domain between YWHAH and Fra-1, we constructed vectors expressing Fra-1 3-segment domains D1 (1–107 aa), D2 (1–127 aa) and D3 (107–271 aa) with HA tags (Fig. 1C), and conducted IP experiments to verify the domain of interaction. The results showed that YWHAH interacted with full length Fra-1 and all three segment domains; however, the interaction between YWHAH and D2 (1–127 aa) was stronger (Fig. 1D), suggesting that the Bag domain of Fra-1 plays an important role in the interaction between the two proteins. In conclusion, we identified YWHAH as a interacting protein of Fra-1 in SGC7901 GC cells.

### YWHAH positively regulates the mRNA and protein expression of Fra-1

**Figure 2:**
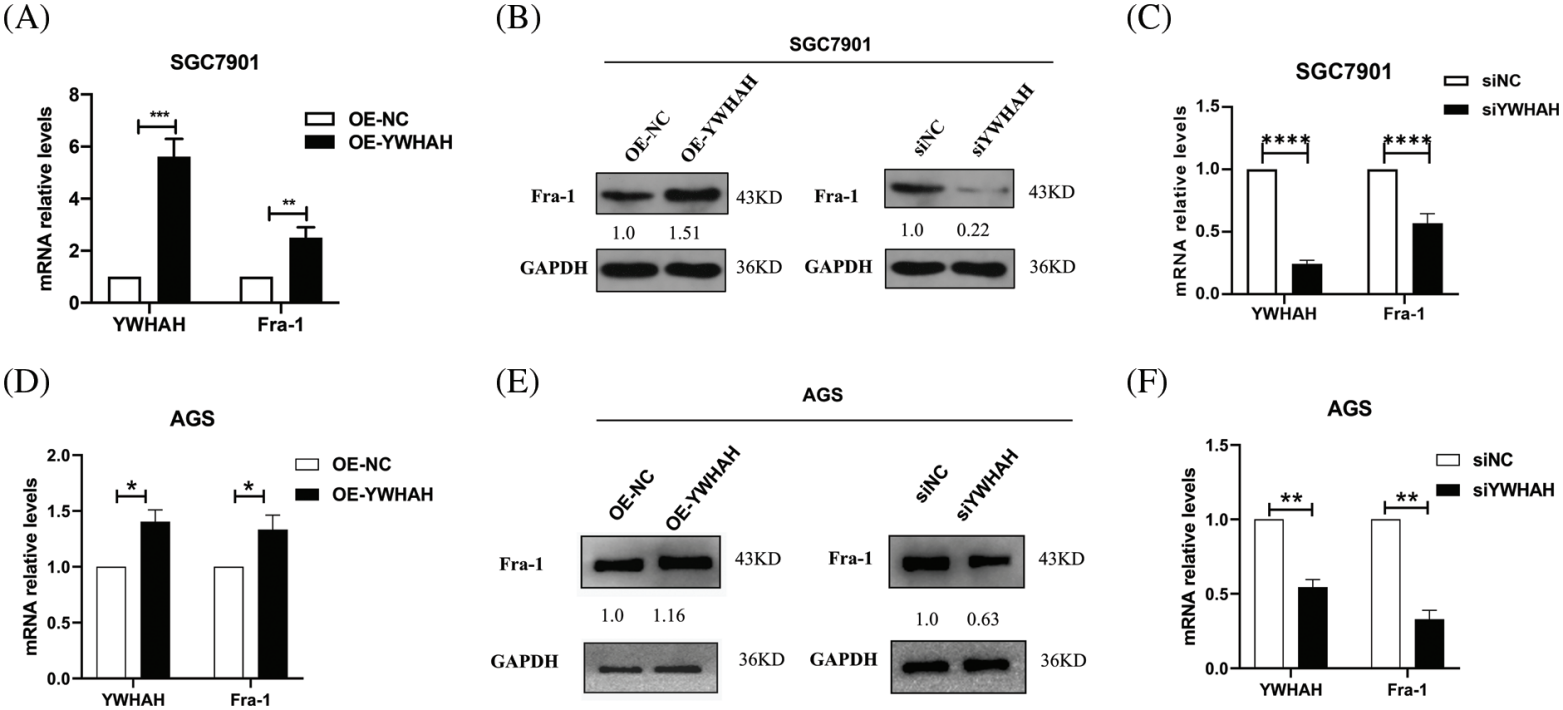
YWHAH positively regulates the mRNA and protein expression of Fra-1. (A) In SGC7901 GC cells, the effect of overexpression of YWHAH on the expression of Fra-1 mRNA was detected using qRT-PCR. (B) In SGC7901 GC cells, the effect of overexpression/interference of YWHAH on the level of the Fra-1 protein was detected using western blotting. (C) In SGC7901 GC cells, the mRNA level of Fra-1 after interference with YWHAH was detected using qRT-PCR. (D) In AGS GC cells, the effect of overexpression of YWHAH on the expression of Fra-1 mRNA was detected using qRT-PCR. (E) Western blotting was used to detect the effect of overexpression/interference of YWHAH on the level of the Fra-1 protein in AGS GC cells. (F) In AGS GC cells, the mRNA level of Fra-1 after interference with YWHAH was detected using qRT-PCR. GAPDH was the internal control, NS: not significant, **p < 0.01, ***p < 0.001. Three independent experiments were conducted.

**Supplementary Figure 1:**
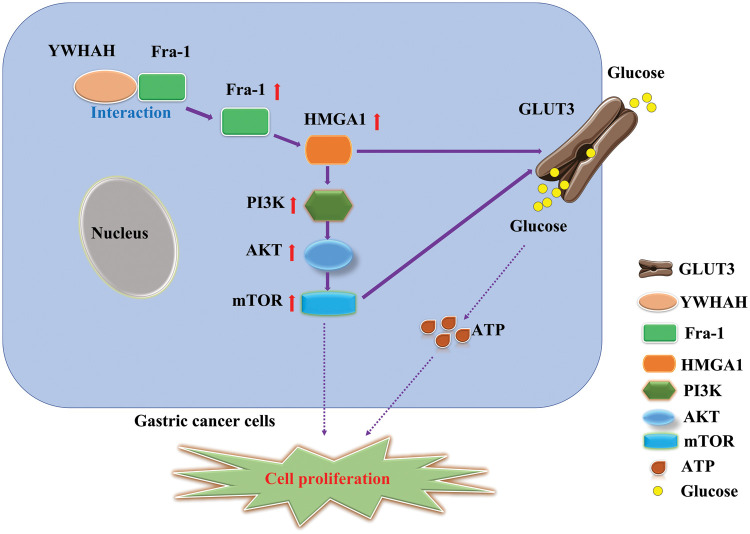
Identification of gene knockout effect of YWHAH siRNA sequence. Three siRNA sequences were designed for the YWHAH gene and transiently transfected into SGC7901 cells. The knockout effects of the three YWHAH siRNA sequences were detected by western blot. The siRNA sequences of si-YWHAH-01, si-YWHHAH-02, and si-YWHHAH-03 were shown from left to right in the figure. Western blot analysis showed that si-YWHAH-01 had the best effect on reducing YWHAH protein level.

To clarify the mutual regulation relationship between YWHAH and Fra-1, we first designed three siRNA sequences for the *YWHAH* gene, and transiently transfected the three siRNAs into SGC7901 GC cells. Western blotting showed that si-YWHAH-01 had the best effect in reducing the protein level of YWHAH (Suppl. Fig. 1); therefore, si-YWHAH-01 was used in subsequent experiments. Then, we overexpressed and silenced *YWHAH*, separately, in SGC7901 GC cells. Western blotting and qRT-PCR assays showed that the mRNA and protein levels of Fra-1 were upregulated after *YWHAH* overexpression (Figs. 2A and 2B) but downregulated after *YWHAH* silencing (Figs. 2B and 2C), suggesting that YWHAH further affected the translation level after regulating the transcription of *Fra-1*. To further verify our experimental results, we repeated the above experiments in AGS cells, and the results were consistent with those in SGC7901 cells (Figs. 2D–2F). The above results showed that YWHAH interacted with and positively regulated Fra-1, and affected its protein level by regulating *Fra-1* transcription.

### YWHAH affects the proliferation of GC cells by positively regulating Fra-1

**Figure 3:**
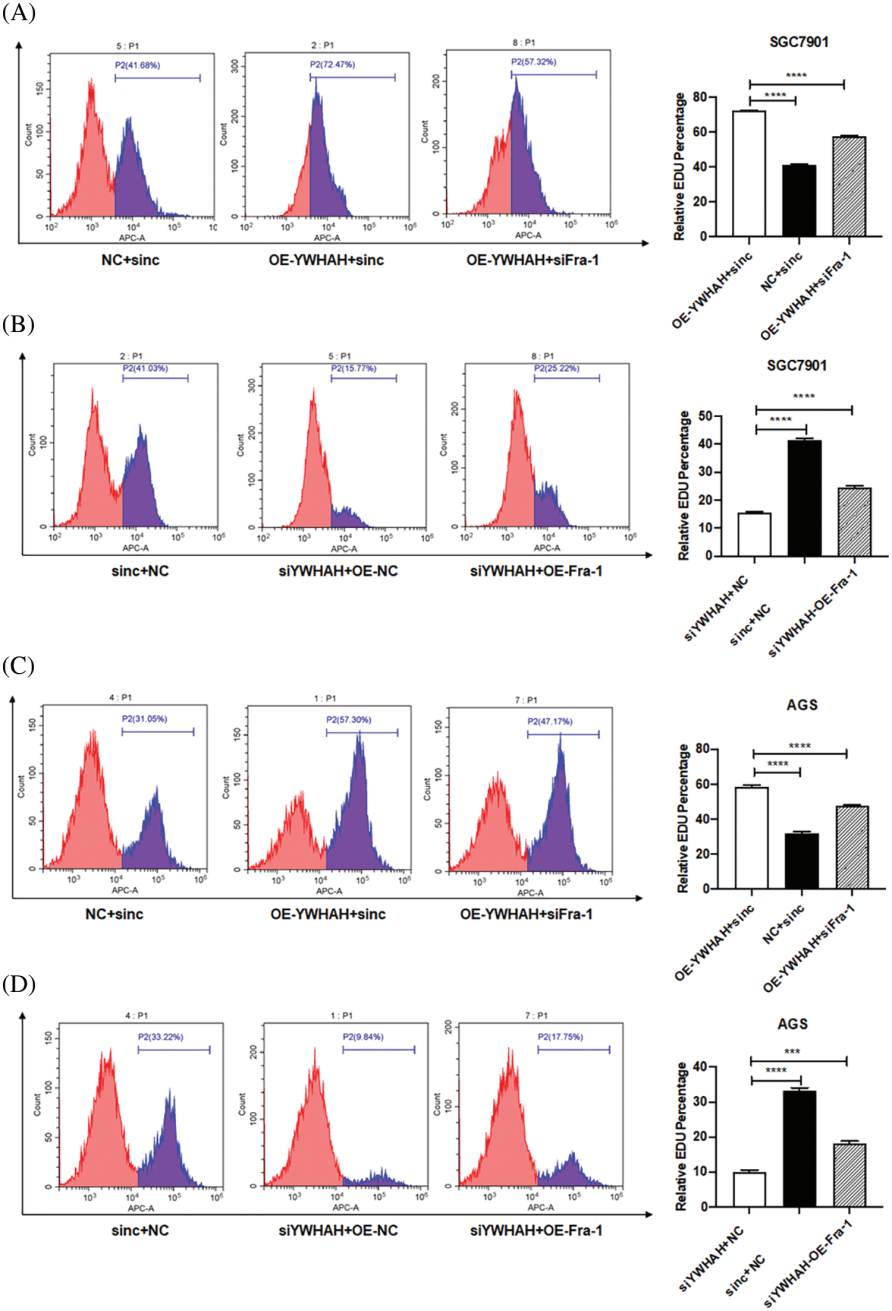
YWHAH affects the proliferation of GC cells by positively regulating Fra-1. (A) YWHAH+siNC, NC+siNC, and YWHAH+siFra-1 plasmids were transfected into SGC7901 GC cells, separately. Cell proliferation was detected using an EdU-647 cell proliferation assay combined with flow cytometry. (B) siYWHAH+ NC, siNC + NC, and siYWHAH+Fra-1 plasmids were transfected into SGC7901 GC cells, separately. Cell proliferation was detected using an EdU-647 cell proliferation assay combined with flow cytometry. (C) YWHAH+siNC, NC+siNC, and YWHAH+siFra-1 plasmids were transfected into AGS GC cells, separately. Cell proliferation was detected using an EdU-647 cell proliferation assay combined with flow cytometry. (D) siYWHAH+ NC, siNC + NC, and siYWHAH+Fra-1 plasmids were transfected into AGS GC cells, separately. Cell proliferation was detected using an EdU-647 cell proliferation assay combined with flow cytometry. ***p < 0.001. Three independent experiments were conducted.

Based on the discovery that YWHAH interacts with and positively regulates the mRNA and protein levels of Fra-1, we assessed whether YWHAH could affect the proliferation of GC cells by regulating Fra-1. Cell proliferation assays combined with flow cytometry showed that the cell proliferation increased after the stable overexpression of *YWHAH* in SGC7901 GC cells. However, when *Fra-1* was silenced in SGC7901 cells stably overexpressing *YWHAH*, cell proliferation was inhibited (Fig. 3A), suggesting that YWHAH might affect GC cell proliferation by regulating Fra-1. When we silenced with *YWHAH* in SGC7901 cells, their proliferation ability was inhibited. However, in cell silenced for *YWHAH*, *Fra-1* overexpression restored cell proliferation (Fig. 3B). The above results showed that YWHAH affected the proliferation of GC cells by regulating Fra-1. To further verify our experimental results, we repeated the above experiments in AGS cells, and the results were consistent with those in SGC7901 cells (Figs. 3C and 3D).

### Whole proteome analysis suggests that Fra-1 affects the activity of the PI3K/AKT/mTOR signal pathway in GC cells

**Figure 4:**
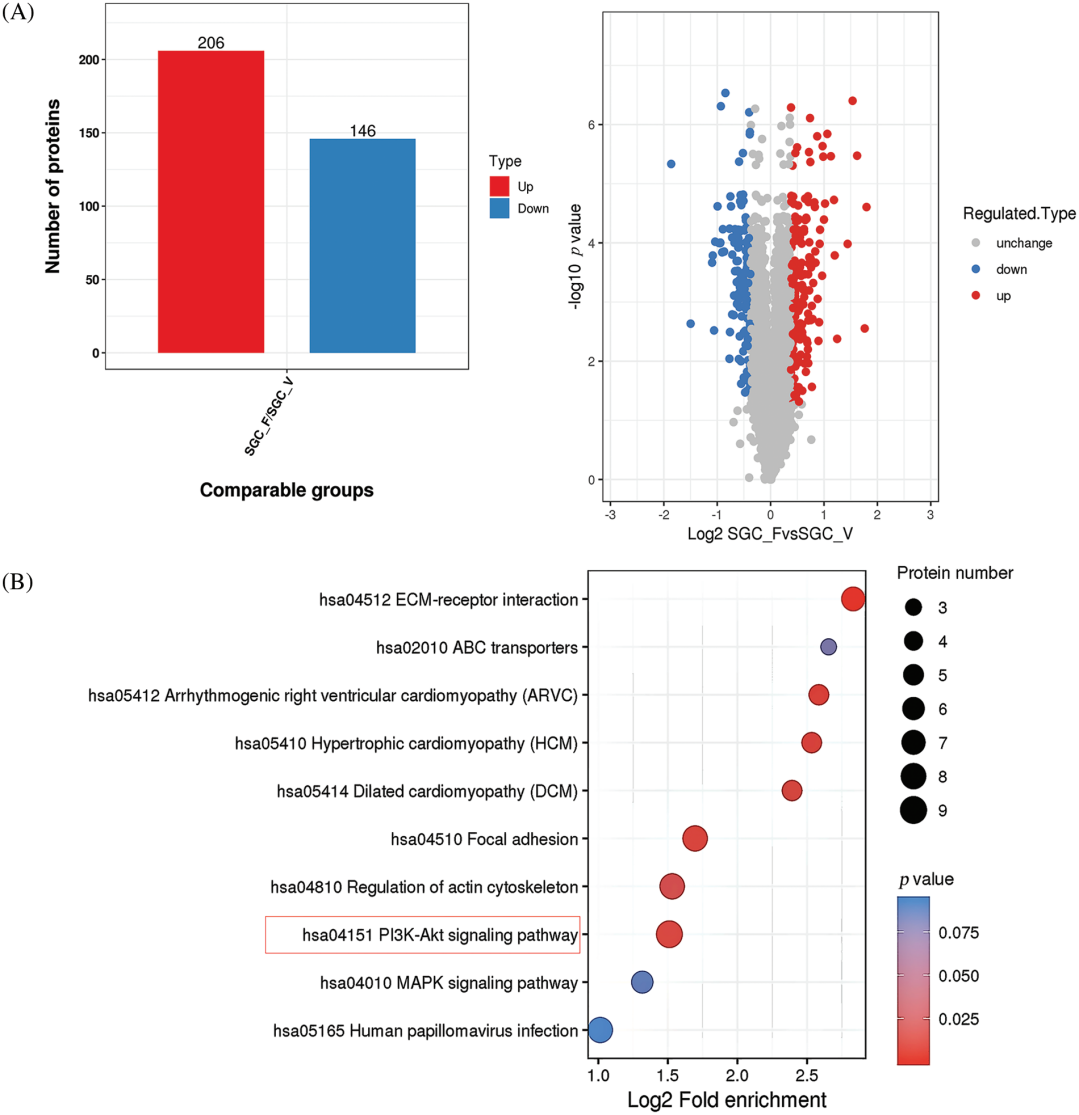
Whole proteome analysis suggests that Fra-1 affects the activity of the PI3K/AKT/mTOR signal pathway in GC cells. (A) Left: quantity distribution diagram of differentially abundant proteins between the Fra-1 overexpression group and the empty vector group of SGC7901 GC cells (column diagram); Right: quantitative volcano map of differentially abundant proteins between the Fra-1 overexpression group and empty vector group in SGC7901 GC cells. The upregulated differentially abundant proteins are represented by red dots in the map, and the downregulated differentially abundant proteins are represented by blue dots. (B) KEGG enrichment analysis of differentially abundant proteins: the vertical axis is the pathway, and the horizontal axis is the log2 converted value of the ratio of the differentially abundant protein in this functional type compared with the ratio of the identified protein. The color of the circle indicates the enrichment significance *p* value, and the size of the circle indicates the number of differentially abundant proteins in the functional class or pathway.

To explore the possible mechanism by which YWHAH regulates Fra-1 to affect the GC cell proliferation, the whole proteome was analyzed after vector (blank control) and Fra-1 plasmids were transfected into SGC7901 GC cells, separately. The results showed that 206 protein molecules were upregulated (Suppl. Table 2) and 146 proteins were downregulated after overexpression of *Fra-1* in GC cells SGC7901 (Fig. 4A, Suppl. Table 3). Based on the analysis of these differentially abundant proteins, we performed functional classification, functional enrichment, and cluster analysis for all differentially abundant proteins. Kyoto Encyclopedia of Genes and Genomes (KEGG) enrichment analysis of upregulated proteins showed that the PI3K/AKT signaling pathway, the mitogen activated protein kinase (MAPK) signal transduction pathway, and the human papillomavirus infection signal transduction pathway were three main signal transduction pathways affected by Fra-1 (Fig. 4B). It has been reported that Fra-1 regulates the expression of HMGA1, and HMGA1 is a regulator of the PI3K/AKT/mTOR signaling pathway. Combined with our previous research results, we clarified whether YWHAH could promote the proliferation of GC cells by positively regulating Fra-1 to activate the HMGA1/PI3K/AKT/mTOR signaling pathway.

### YWHAH activates the HMGA1/PI3K/AKT/mTOR signaling pathway by positively regulating Fra-1 to affect the proliferation of gastric cancer cells

**Figure 5:**
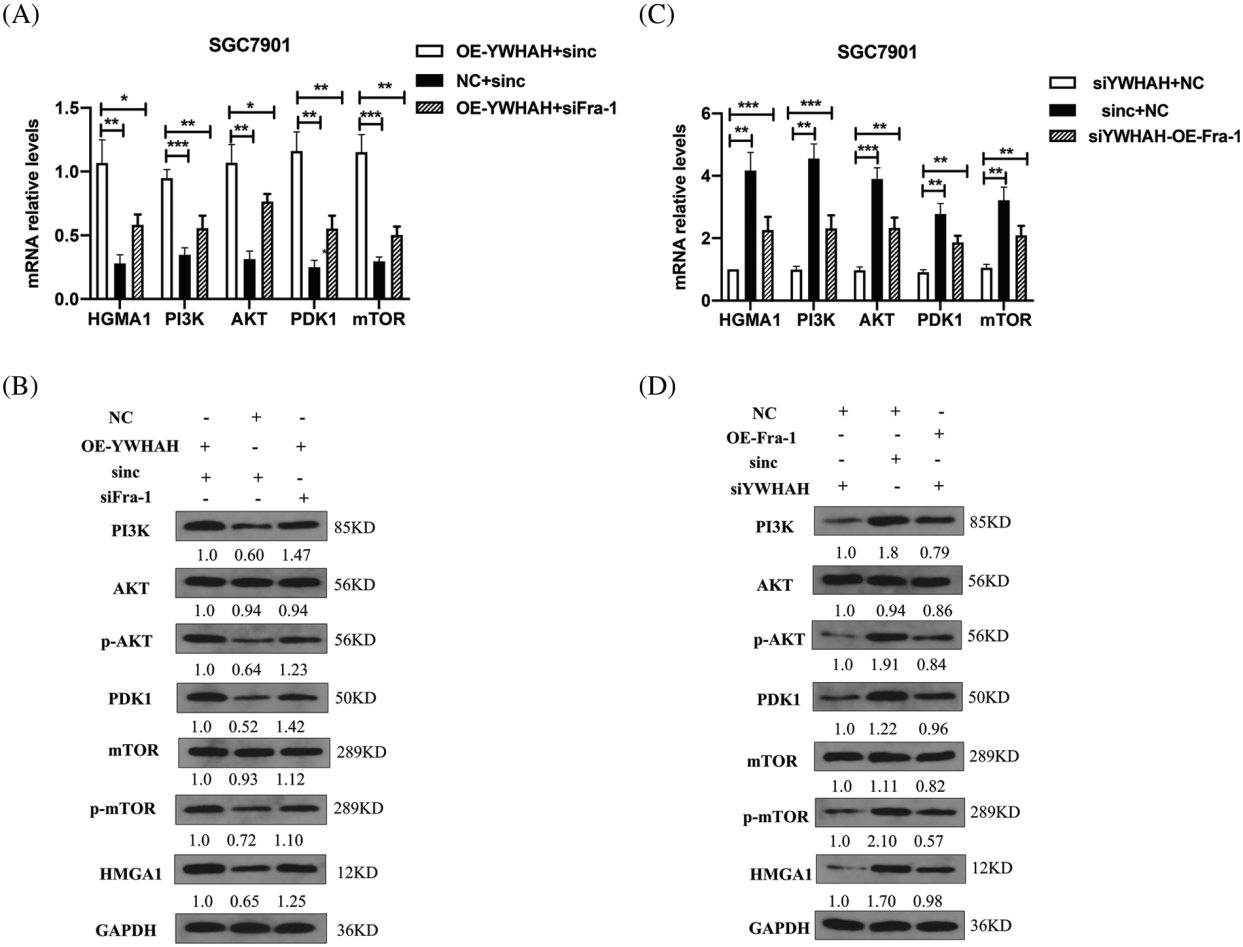
YWHAH regulates the activity of the HMGA1/PI3K/AKT/mTOR signaling pathway through Fra-1. (A and C) The mRNA levels of HMGA1/PI3K/AKT/mTOR signal pathway-related molecules were detected by qRT-PCR. (B and D) Western blotting detection of the protein level of HMGA1/PI3K/AKT/mTOR signaling pathway-related molecules. NS means: meaningless, *p < 0.05, **p < 0.01, ***p < 0.001, *** *p* < 0.0001. Internal reference: GAPDH, three independent repeated experiments.

To confirm whether YWHAH can affect the proliferation of GC cells by positively regulating Fra-1 to activate the HMGA1/PI3K/AKT/mTOR signaling pathway, we first transfected NC+siNC, OE-YWHAH+siNC, and OE-YWHAH+siFra-1 plasmids into SGC7901 GC cells, and detected the mRNA levels of PI3K/AKT/mTOR signal pathway related molecules (*PI3K*, *AKT*, *PDK1*, *MTOR*, etc.) using qRT-PCR. The results showed that the mRNA expression levels of *PI3K*, *AKT*, *PDK1*, and *MTOR* were upregulated after overexpression of *YWHAH*. However, after *Fra-1* silencing in a background of *YWHAH* overexpression, upregulation of the mRNA expression levels of *PI3K*, *AKT*, *PDK1*, and *MTOR* molecules was inhibited (Fig. 5A). In addition, western blotting analysis also showed that the protein levels of PI3K and PDK1 were upregulated, as were the levels of phosphorylated (p)-AKT and p-mTOR after *YWHAH* overexpression. However, after *Fra-1* silencing in a background of *YWHAH* overexpression, the upregulation of PI3K and PDK1 protein levels was inhibited, as were the upregulation of p-AKT and p-mTOR levels (Fig. 5B). The results suggest that YWHAH can activate the PI3K/AKT/mTOR signaling pathway through Fra-1.

At the same time, we silenced the expression of *YWHAH*, in which siNC+NC, siYWHAH+NC, and siYWHAH+OE-Fra-1 plasmids were transfected into SGC7901 GC cells, and the related molecules were detected using qRT-PCR. The results showed that the mRNA expression levels of *PI3K*, *AKT*, *PDK1*, and *MTOR* were downregulated after *YWHAH* silencing. However, overexpression of *Fra-1* in a background of *YWHAH* silencing restored the mRNA expression levels of *PI3K*, *AKT*, *PDK1*, and *MTOR* (Fig. 5C). In addition, the protein levels of PI3K/AKT/mTOR signaling pathway-related molecules were detected by western blotting. The results showed that after *YWHAH* silencing, the protein levels of PI3K and PDK1 were downregulated, and the levels of p-AKT and p-mTOR were downregulated. Overexpression of *Fra-1* in a background of *YWHAH* silencing restored the protein levels of PI3K and PDK1, as well as the levels of p-AKT and p-mTOR (Fig. 5D). Our results suggested that YWHAH can promote the proliferation of gastric cancer cells by activating the HMGA1/PI3K/AKT/mTOR signaling pathway through Fra-1.

**Figure 6:**
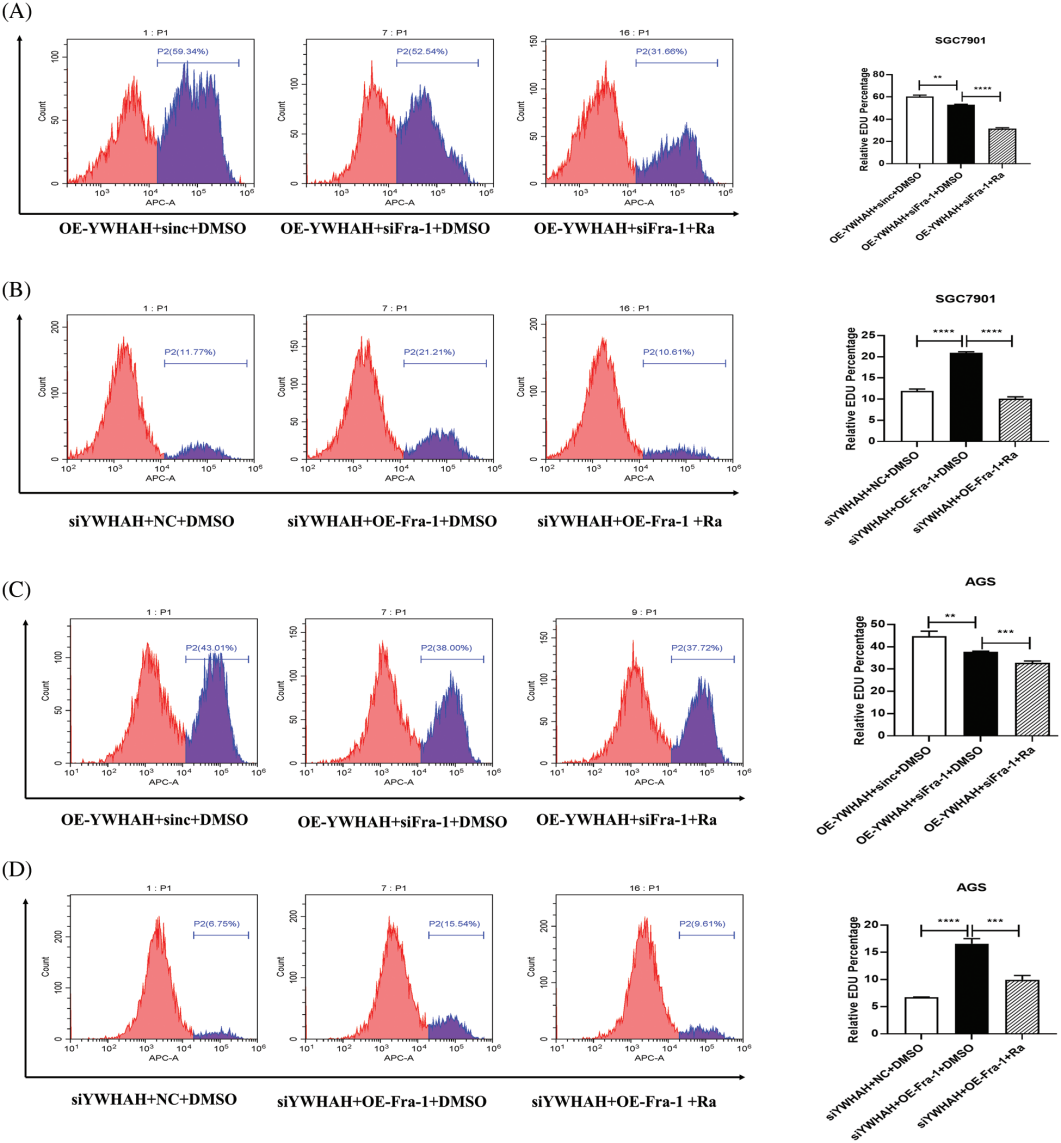
YWHAH promotes gastric cancer cell proliferation by activating the PI3K/AKT/mTOR signaling pathway. (A) Using EdU-647 cell proliferation assay combined with flow cytometry to detect the proliferation level of SGC7901 GC cells in three groups: OE-YWHAH+siNC+DMSO, OE-YWHAH+siFra-1+DMSO, OE-YWHAH+siFra-1+Rapamycin. (B) Using EdU-647 cell proliferation assay combined with flow cytometry to detect the proliferation level of SGC7901 GC cells in three groups: siYWHAH+NC+DMSO, siYWHAH+OE-Fra-1+DMSO, and siYWHAH+OE-Fra-1+Rapamycin. (C) Using EdU-647 cell proliferation assay combined with flow cytometry to detect the proliferation level of AGS GC cells in three groups: OE-YWHAH+siNC+DMSO, OE-YWHAH+siFra-1+DMSO, OE-YWHAH+siFra-1+Rapamycin. (D) Using EdU-647 cell proliferation assay combined with flow cytometry to detect the proliferation level of AGS GC cells in three groups: siYWHAH+NC+DMSO, siYWHAH+OE-Fra-1+DMSO, and siYWHAH+OE-Fra-1+Rapamycin. **p < 0.01, ***p < 0.001, ****p < 0.0001. Three independent experiments were conducted.

To further confirm that YWHAH promotes gastric cancer cells proliferation by activating the PI3K/AKT/mTOR signaling pathway, we used rapamycin, an inhibitor of the PI3K/AKT/mTOR signaling pathway to treat gastric cancer cells. Firstly, we divided gastric cancer cells SGC7901 into three groups: OE-YWHAH+siNC+DMSO, OE-YWHAH+siFra-1+DMSO, OE-YWHAH+siFra-1+Rapamycin by transfecting the different plasmids and whether treating cells with rapamycin. Then, we tested the gastric cancer cells proliferation using an EDU proliferation detection kit and flow cytometry. Our results showed that compared to the OE-YWHAH+siNC+DMSO group, OE-YWHAH+siFra-1+DMSO group had a decrease in the proportion of proliferating cells, OE-YWHAH+siFra-1+Rapamycin group had a more significant reduction in the proportion of proliferating cells (Fig. 6A). To further confirm the effect of silencing YWHAH, we divided gastric cancer cells SGC7901 into three groups: siYWHAH+NC+DMSO, siYWHAH+OE-Fra-1+DMSO, siYWHAH+OE-Fra-1+Rapamycin by transfecting the different plasmids and whether treating cells with rapamycin. Then we tested the gastric cancer cells proliferation using an EDU proliferation detection kit and flow cytometry. Our results showed that compared to the siYWHAH+NC+DMSO group, the siYWHAH+OE-Fra-1+DMSO group had an increase in the proportion of proliferating cells. But, the siYWHAH+OE-Fra-1+Rapamycin group had a decrease in the proportion of proliferating cells (Fig. 6B). In AGS cells, we obtained results with the same trend (Figs. 6C and 6D). Above of all, our results suggested that YWHAH could promote the proliferation of gastric cancer cells by activating the PI3K/AKT/mTOR signaling pathway.

## Discussion

The pathogenesis of GC is complex. Although some progress has been made in its early diagnosis, surgical resection, and adjuvant chemotherapy, GC is still difficult to identify at the early stage of onset. The 5-year survival rate of patients with GC is not high. GC is one of the most aggressive cancers of the human digestive system [30–33]. Previous studies have shown that the pathogenesis of GC is closely related to *Helicobacter pylori* infection, proto oncogene activation, and tumor suppressor gene inactivation [6–8,12–14,34]; however, the detailed mechanism is unclear. More experimental evidence is needed to clarify its pathogenesis and potential molecular targets, to provide an experimental basis for early GC diagnosis, the identification of potential molecular therapeutic targets, and prognosis prediction.

Fra-1 is a member of the FOS family and is an important nuclear transcription factor that regulates the growth, differentiation, and apoptosis of normal cells [6,7,19–21]. Fra-1 is highly expressed in a variety of malignant tumors and plays an important role in cell transformation, proliferation, invasion, and metastasis [18–21,35]. Our previous study found that Fra-1 was highly expressed in GC tissues compared with that in adjacent non-cancerous tissues. *In vitro* experiments confirmed that *Fra-1* overexpression inhibited GC cell apoptosis and increased the proportion of S-phase cells [6,36]. To further clarify the role and possible mechanism of Fra-1 in GC cell proliferation, we identified and confirmed YWHAH as a Fra-1-interacting protein in GC cells. Protein interaction is widely involved in cell-cell interactions, metabolic and developmental controls and other biological processes [37,38]. Protein interaction also plays a key role in predicting the function of target proteins [39,40]. We further studied the function of YWHAH, which provided a new perspective on the role and possible mechanism of Fra-1 in GC.

Based on the discovery that YWHAH interacts with Fra-1, further qRT-PCR and western blotting experiments showed that YWHAH positively regulates the transcription of *Fra-1* and then affects the protein level of Fra-1. In addition, *YWHAH* overexpression in SGC7901 GC cells enhanced their proliferation. Compared with the vector group, cell proliferation was inhibited in the group transfected with YWHAH and siFra-1. The results of silencing *YWHAH* were opposite to those of *YWHAH* overexpression. These results suggested that YWHAH promotes GC cell proliferation by activating Fra-1. YWHAH is a 14-3-3 eta protein and a member of the 14-3-3 protein family [41,42]. A remarkable feature of 14-3-3 proteins is that they can bind to a variety of signal proteins with diverse functions, including kinases, phosphatases, and transmembrane receptors [43,44]. 14-3-3 proteins play an important role in biological processes, such as mitotic signal transduction, apoptotic cell death, and cell cycle control [45,46]. Wu et al. found that the miR-660-5p/YWHAH axis could activate the PI3K/AKT pathway to promote epithelial mesenchyme transition and the cell cycle process in hepatoma cells [47]. In thyroid cancer, the long noncoding RNA *MAPKAPK5-AS1* promoted the proliferation and migration of thyroid cancer cell lines by targeting miR-519e-5p/YWHAH [48]. Our results provide new ideas and an experimental basis to clarify the role and possible mechanism of YWHAH in malignant tumors.

**Figure 7:**
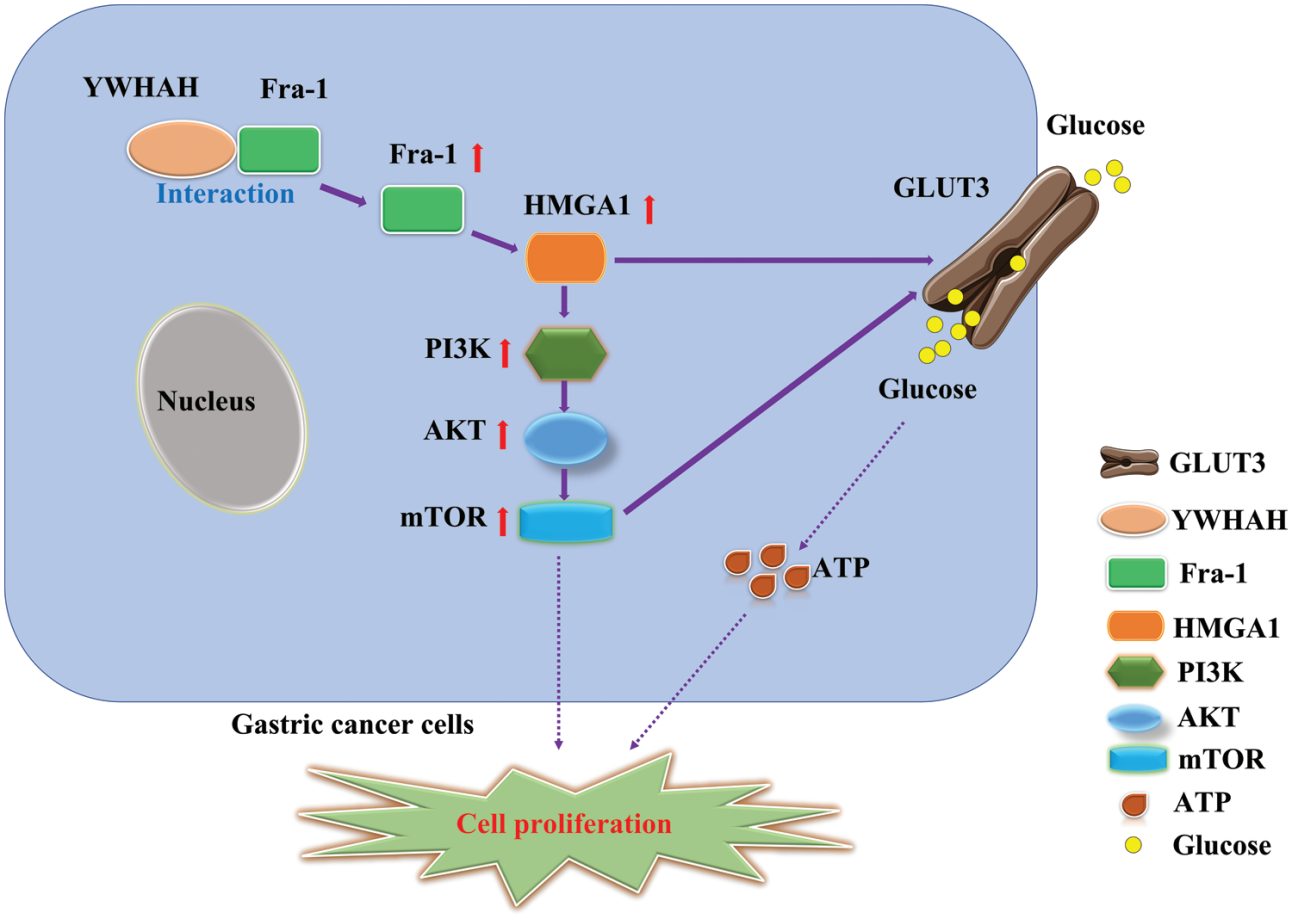
Schematic diagram of the effect of YWHAH interacting with Fra-1 on the proliferation of gastric cancer cells.

To further explore the specific mechanism by which YWHAH affects GC cell proliferation via Fra-1, we overexpressed *Fra-1* in SGC7901 GC cells for whole proteome analysis. KEGG enrichment analysis of all differentially abundant proteins showed that the PI3K/AKT signaling pathway was abnormally activated. Then, we confirmed that YWHAH promoted GC cell proliferation by positively regulating Fra-1 to activate the HMGA1/PI3K/AKT/mTOR signaling pathway using western blotting, flow cytometry, and other technologies (Fig. 7). The PI3K/AKT signaling pathway plays an important role in basic intracellular functions, such as cell growth, apoptosis, translation, and cell metabolism [49–51]. It is important not only in carcinogenesis, but also in identifying potential new therapeutic targets [52–54]. The PI3K/AKT pathway is stimulated by receptor tyrosine kinases and by cytokine receptor activation. Tyrosine residues are subsequently phosphorylated and provide anchor sites for PI3K translocation to the membrane, thus participating in the transduction of various extracellular matrix molecules and cytokines [55,56]. Other studies have shown that changes in the activity of the PI3K/AKT/mTOR signaling pathway affect the proliferation of tumor cells [57–59]. Our results also confirmed that activation of the PI3K/AKT/mTOR signaling pathway promoted GC cell proliferation, which was consistent with the above related research results.

The experimental results provide new molecular targets to explore the pathogenesis of GC and provide an experimental basis to clarify the pathogenesis of GC.

## Data Availability

The datasets used and/or analyzed during the current study are available from the corresponding author on reasonable request.

## Supplementary Materials

**Supplementary Table 1:** Analysis of flag lane specific protein molecules by mass spectrometry (25~40 kDa) (Top 25)

Number	Gene	Gene (official full name)	Score
1	PSMA4	Proteasome subunit alpha type (Fragment)	503.72
2	YWHAE	14-3-3 protein epsilon	294.00
3	C1QBP	Complement component 1 Q subcomponent-binding protein, mitochondrial	240.54
4	GSTO1	Glutathione S-transferase omega-1 (Fragment)	213.95
5	PLPBP	Pyridoxal phosphate homeostasis protein	199.19
6	WDR77	Methylosome protein 50	192.08
7	CLIC1	Chloride intracellular channel protein 1	189.42
8	ALDOA	Fructose-bisphosphate aldolase	175.86
9	PGAM5	Serine/threonine-protein phosphatase PGAM5, mitochondrial	167.61
10	PPP1CA	Serine/threonine-protein phosphatase PP1-alpha catalytic subunit	147.59
11	YWHAH	14-3-3 protein eta	143.73
12	SPIN1	Spindlin-1	128.19
13	YWHAG	14-3-3 protein gamma	124.87
14	LDHB	L-lactate dehydrogenase B chain	121.86
15	CLNS1A	Methylosome subunit pICln	113.38
16	ATP5F1C	ATP synthase subunit gamma, mitochondrial	112.78
17	YWHAB	14-3-3 protein beta/alpha	109.02
18	EEF1B2	Elongation factor 1-beta	106.00
19	TPI1	Triosephosphate isomerase	100.97
20	IGKV2-40	Immunoglobulin kappa variable 2–40	91.42
21	LDHA	L-lactate dehydrogenase A chain	85.12
22	RPS3A	40S ribosomal protein S3a	79.72
23	PCNA	Proliferating cell nuclear antigen	66.47
24	YWHAZ	14-3-3 protein zeta/delta	62.13
25	YWHAQ	14-3-3 protein theta	54.95

**Supplementary Table 2:** Up-regulated protein molecules after overexpression of Fra-1 in gastric cancer cells (Top 100)

Number	Gene name	Ratio	Number	Gene name	Ratio
1	ITSN2	3.474	51	SYNE2	1.600
2	IQCB1	3.392	52	MAGEE1	1.598
3	MED18	3.074	53	RNF149	1.593
4	CYP26B1	2.899	54	MORC2	1.587
5	HMGA2	2.717	55	SPATA31E1	1.580
6	CYSRT1	2.372	56	CEP152	1.568
7	ASXL2	2.299	57	CMYA5	1.564
8	HIST1H1C	2.277	58	CA13	1.558
9	PLIN2	2.187	59	ABLIM1	1.554
10	SNRNP27	2.088	60	OCIAD2	1.55
11	G2E3	2.027	61	ZNF512	1.547
12	SERPINB7	1.999	62	MBD4	1.544
13	ANPEP	1.980	63	RYR3	1.543
14	C18orf63	1.963	64	HIST1H1D	1.535
15	HSP90AA4P	1.952	65	PLIN4	1.531
16	NEB	1.900	66	S100A2	1.530
17	PFN2	1.891	67	EPS8	1.512
18	ABCG2	1.878	68	ITGAE	1.511
19	HIST1H1E	1.861	69	CGAS	1.509
20	SMPD2	1.842	70	FPGS	1.508
21	TBC1D4	1.831	71	A1BG	1.505
22	ADAM19	1.792	72	KANK1	1.504
23	CPA4	1.785	73	EIF2AK4	1.497
24	AARSD1	1.776	74	CDC123	1.496
25	NIPSNAP3A	1.766	75	OFD1	1.493
26	C17orf80	1.746	76	RAD23A	1.486
27	ABCC8	1.739	77	DOPEY1	1.483
28	ANGPT1	1.725	78	CEP350	1.478
29	PABPN1L	1.710	79	RPS29	1.477
30	C1orf50	1.709	80	IMUP	1.477
31	PRORY	1.708	81	COPE	1.477
32	COL4A1	1.691	82	SDC4	1.474
33	TPBG	1.684	83	NFXL1	1.472
34	SERPINI2	1.679	84	C2CD2	1.467
35	DLEU7	1.675	85	WASH6P	1.465
36	CCDC36	1.668	86	FBN1	1.464
37	ZNF185	1.653	87	HSPA1L	1.462
38	EXO5	1.651	88	MAPK7	1.452
39	KIAA1468	1.651	89	GOLGA8R	1.452
40	ITGA9	1.645	90	DYNLT3	1.447
41	RPP38	1.642	91	DTWD1	1.445
42	ACTL8	1.635	92	IRS2	1.445
43	EPX	1.632	93	C9orf40	1.441
44	SGCD	1.632	94	SYAP1	1.436
45	ID1	1.630	95	MLLT3	1.435
46	NPAT	1.627	96	MYOD1	1.435
47	LRRCC1	1.625	97	MKL2	1.430
48	ZBTB10	1.623	98	ELF1	1.426
49	SKA3	1.612	99	FRYL	1.423
50	OASL	1.602	100	VPS53	1.423

**Supplementary Table 3:** Down-regulated protein molecules after overexpression of Fra-1 in gastric cancer cells (Top 100)

Number	Gene name	Ratio	Number	Gene name	Ratio
1	ALPI	0.275	51	DHCR7	0.672
2	EPB41L3	0.354	52	ITPR1	0.673
3	SSR3	0.468	53	TFRC	0.674
4	MTRF1	0.472	54	GLO1	0.675
5	SLC2A3	0.48	55	ASS1	0.677
6	PDE1A	0.487	56	SPANXB1	0.68
7	MRPS23	0.502	57	HIST1H2AJ	0.682
8	PLCL2	0.511	58	SIAE	0.684
9	S100P	0.52	59	BDH1	0.687
10	TSPO	0.521	60	EZH2	0.692
11	ALPP	0.524	61	ATP6V0D1	0.698
12	HMG20B	0.533	62	COX11	0.7
13	MRPS17	0.538	63	MAPKAPK5	0.7
14	GPNMB	0.547	64	PCK2	0.7
15	METTL7A	0.556	65	NECTIN2	0.701
16	NCKAP1L	0.586	66	FILIP1	0.703
17	NCBP2	0.587	67	GUSB	0.703
18	ENO3	0.59	68	FBXO4	0.705
19	MRC2	0.593	69	CADM1	0.705
20	WASL	0.598	70	FAU	0.706
21	COG3	0.607	71	SGMS1	0.707
22	MRPS2	0.607	72	CMC2	0.708
23	HEBP1	0.608	73	GNPTG	0.713
24	AGA	0.62	74	CLTB	0.713
25	SRRM1	0.622	75	LANCL1	0.715
26	YIPF5	0.625	76	N4BP3	0.718
27	TUSC3	0.63	77	SLC39A10	0.719
28	CLTA	0.63	78	TP53I3	0.721
29	STEAP4	0.64	79	ISCU	0.723
30	RBM3	0.643	80	NNT	0.723
31	SLC25A6	0.644	81	SUMF1	0.723
32	INTS5	0.646	82	CSRP2	0.724
33	SLC25A4	0.648	83	ALDH5A1	0.724
34	MRPL18	0.65	84	FAHD1	0.725
35	CD63	0.652	85	TMEM11	0.726
36	FAR1	0.653	86	MEIS3P1	0.727
37	TRIM29	0.656	87	PDE3A	0.727
38	CYBRD1	0.657	88	SNRPD3	0.728
39	GGCX	0.659	89	EXOG	0.728
40	TEAD1	0.661	90	MTX2	0.729
41	COQ7	0.661	91	SUMF2	0.73
42	YIPF3	0.661	92	ARMCX3	0.731
43	ZBED1	0.662	93	GDA	0.732
44	CPS1	0.664	94	MSLN	0.733
45	AKAP4	0.666	95	MP68	0.733
46	MRPL35	0.667	96	KLHL13	0.734
47	ALDH6A1	0.667	97	S100A4	0.734
48	VKORC1	0.669	98	ARGLU1	0.735
49	ATP5S	0.669	99	ISOC2	0.736
50	RBX1	0.671	100	RHOB	0.736
